# Supplementary material for: The Systems Biology Research Tool: evolvable open-source software
Source: BMC Syst Biol. 2008 Jun 29;2:55. doi: 10.1186/1752-0509-2-55 (PMC2446383; doi:10.1186/1752-0509-2-55)
Supplement: Additional file 1 — SBRT Archive. An archive of the current version of the Systems Biology Research Tool. [file 1752-0509-2-55-S1.zip › sbrt-1.4.0/doc/developers_guide/api/sbrt/kernel/math/QuadraticExpr.Term.html]

QuadraticExpr.Term


|  |  |  |
| --- | --- | --- |
| **Overview** \| **Package** \| **Class** \| **Use** \| **Tree** \| **Deprecated** \| **Index** \| **Help** | |  |
| **PREV CLASS**   **NEXT CLASS** | **FRAMES**    **NO FRAMES**     **All Classes** |  |
| SUMMARY: NESTED \| FIELD \| CONSTR \| METHOD | DETAIL: FIELD \| CONSTR \| METHOD |  |


---


## sbrt.kernel.math Interface QuadraticExpr.Term<V>

**Type Parameters:**: `V` - the variable type.

**All Superinterfaces:**: java.lang.Iterable<V>, Polynomial.Term<V>

**Enclosing interface:**: QuadraticExpr<V>

---

``` public static interface QuadraticExpr.Term<V> extends Polynomial.Term<V> ```

This interface is used to represent the terms of quadratic expressions.
It has not been fully implemented.

**Author:**
:   This interface was written and documented by Jeremiah Wright
    while in the Wagner lab.

---

**Method Summary**

**Methods inherited from interface sbrt.kernel.math.Polynomial.Term**

`equals, getCoefficient, getVariables, hasAllVariables, hashCode, hasVariable, iterator, power, toString, variables`

---


|  |  |  |
| --- | --- | --- |
| **Overview** \| **Package** \| **Class** \| **Use** \| **Tree** \| **Deprecated** \| **Index** \| **Help** | |  |
| **PREV CLASS**   **NEXT CLASS** | **FRAMES**    **NO FRAMES**     **All Classes** |  |
| SUMMARY: NESTED \| FIELD \| CONSTR \| METHOD | DETAIL: FIELD \| CONSTR \| METHOD |  |


---
